# Serum lactate poorly predicts central venous oxygen saturation in critically ill patients: a retrospective cohort study

**DOI:** 10.1186/s40560-019-0401-5

**Published:** 2019-09-05

**Authors:** Roshan Bisarya, Deena Shaath, Arman Pirzad, Lewis Satterwhite, Jianghua He, Steven Q. Simpson

**Affiliations:** 1 School of Medicine, University of Kansas, Kansas City, KS USA; 2 Department of Internal Medicine, University of Kansas, Kansas City, KS USA; 3 Division of Pulmonary and Critical Care Medicine, University of Kansas, 3901 Rainbow Boulevard, Mail Stop 3047, Kansas City, KS 66160 USA; 4 Department of Biostatistics, University of Kansas, Kansas City, KS USA

**Keywords:** Lactic acid, Shock, Hyperlactatemia, Hypoxia, Sepsis, Septic shock

## Abstract

**Background:**

Serum lactate and central venous oxygen saturation (ScvO_2_) are commonly used and commonly recommended as markers of tissue oxygenation in shock states. Medical literature has both explicitly stated and implied that the two biomarkers are interchangeable in the management of patients with shock. However, there have been relatively few direct comparisons of these tests in clinical circumstances, and the relationship between them is uncertain. The objective of our study was to evaluate whether simultaneous or near-simultaneous measurements of lactate and ScvO_2_ reveal a consistent relationship between these two biomarkers.

**Methods:**

A retrospective cohort study was conducted in an urban, academic US hospital. All adults in ICUs between March 2007 and March 2017 who had a lactate measurement and ScvO_2_ or mixed venous oxygen saturation (SvO_2_) measurement made +/− 1 h from the lactate were included. Linear and non-linear correlations of ScvO_2_ and lactate were assessed in a variety of shock states.

**Results:**

Two thousand sixty-two patients were included. Lactate and ScvO_2_ correlated poorly (*r*^2^ = 0.0041, *p* = 0.0019). This was true for patients with ScvO_2_ ≤ 65% (*r*^2^ = 0.0431, *p* < 0.001), patients with normal kidney and liver function (*r*^2^ = 0.0517, *p* < 0.001), and septic shock patients (*r*^2^ = 0.0037, *p* = 0.17). For patients with an O_2_ extraction ratio ≥ 50%, lactate and ScvO_2_ were strongly correlated (*r*^2^ = 0.93, *p* = 0.0019), but these patients represented only 2.8% of patients in whom the ratio could be calculated.

**Conclusions:**

Lactate can predict ScvO_2_ when patients are at or below the critical oxygen delivery threshold, but relatively few shock patients meet this criterion. In the overall population of critically ill patients, serum lactate predicts ScvO_2_ poorly, even after controlling for factors that may affect lactate production. Lactate and ScvO_2_ should not be assumed to be interchangeable markers of tissue oxygenation/perfusion.

**Electronic supplementary material:**

The online version of this article (10.1186/s40560-019-0401-5) contains supplementary material, which is available to authorized users.

## Background

The critical care literature has considered central venous oxygen saturation (ScvO_2_), mixed venous oxygen saturation (SvO_2_), and serum lactate to be measures of tissue oxygenation in shock [1–9]. Following the Rivers trial of early goal-directed therapy (EGDT) for sepsis, ScvO_2_ became popular as a substitute for SvO_2_ in a variety of shock states [10], and the two measures have been demonstrated to be strongly correlated [6, 8, 11, 12]. A trio of controlled trials subsequently demonstrated that EGDT does not provide a survival advantage in all patients with septic shock, but the studies did not address the utility of ScvO_2_ as a target for resuscitation of septic shock, because half of the patients in these trials had normal ScvO_2_ at the time of randomization [13]. Some authors advocate that ScvO_2_ remains a vital component of sepsis treatment that should not be left out [14]. Serum lactate has also been used as a marker for tissue oxygenation [1–3, 5, 9, 15, 16]; in fact, both ScvO_2_ and lactate are independent predictors of mortality in sepsis, and normalization of either or both biomarkers is associated with improved outcomes in sepsis and septic shock [17–23]. Additionally, both lactate and ScvO_2_ have been used as markers of tissue oxygenation in cardiogenic and hemorrhagic shocks [24–28].

Given their use as markers of tissue oxygenation, it has been posited that levels of serum lactate and ScvO_2_ may be inversely correlated in at least some forms of shock, as ScvO_2_ should be decreased in patients with inadequate tissue perfusion, and inadequate tissue perfusion should result in increased lactate production [8]. It is likely that such an inverse relationship exists below the critical oxygen delivery point in any given patient, but the relationship could be more complex at other levels of oxygen delivery and uptake. In fact, Astiz et al. were unable to determine a level of oxygen delivery or of SvO_2_ that is predictably associated with lactic acidosis in patients with sepsis or acute myocardial infarction [29]. Furthermore, lactate production can be stimulated by alternate mechanisms, and serum lactate can be increased in the absence of tissue hypoxia. We evaluated a large number of patients with shock of various etiologies to examine the relationship between serum lactate and ScvO_2_ and to determine whether a correlation does exist between levels of these two markers in any shock subset.

## Methods

This was a retrospective cohort study performed at The University of Kansas Hospital (TUKH) in Kansas City, Kansas, in accordance with STROBE guidelines for observational studies. The protocol was approved by the Institutional Review Board, with approval number 00001581. All data were obtained from the electronic medical record (Epic, Verona, WI, USA) and collected in Crystal Reports (SAP Software Solutions). All adults (≥ 18 years) in ICUs between March 2007 and March 2017 who had a lactate measurement and measurement of central venous or mixed venous blood gases made ± 1 h from the lactate were included in the study. Previous to August 2014, there was no specific laboratory category in the electronic medical record (EMR) for ScvO_2_, and these measurements were reported by the laboratory as SvO_2_. After August 2014, 15.4% of venous gases were SvO_2_ measures. We assumed similar proportions for gases measured before the ScvO_2_ category became available for reporting, and we combined ScvO_2_ and SvO_2_ measures for analyses but refer to the values as ScvO_2_. For both time periods, we excluded any blood gases characterized only as “venous.”

We recorded age, sex, hospital mortality, length of hospital stay (LOS), length of ICU stay (ICU-LOS), need for dialysis, and need for mechanical ventilation. Discharge diagnoses regarding the type of shock state were recorded based on the International Classification of Diseases ICD-9 or ICD-10 codes and included septic, cardiogenic, traumatic, and shock not otherwise specified (NOS).

The following data were collected within ± 1 day of the patient selection data: creatinine, total bilirubin, aspartate aminotransferase (AST), alanine aminotransferase (ALT), total bilirubin, hemoglobin, and thiamine. We recorded the following medication use in ICU: metformin, epinephrine, norepinephrine, dopamine, and linezolid, which we refer to collectively as lactic acidosis-associated medications. Additionally, using ICD-9 and ICD-10 codes, we determined if the patients had the following pre-existing conditions: chronic kidney disease (CKD), chronic respiratory failure (CRF), chronic liver disease (CLD), chronic hypertension, and type 2 diabetes.

Statistical analysis was performed using Stata version 15.0 (StataCorp, College Station, TX, USA). Simple least squares linear regressions were performed, comparing serum lactate concentration with ScvO_2_ for all eligible patients. We performed secondary regression analyses of lactate and ScvO_2_ for patients meeting the following specific constraints: normal hepatic and renal function (AST ≤ 40 U/L or ALT ≤ 50 U/L and creatinine ≤ 1.2 mg/dL), septic shock, low ScvO_2_ (≤ 65%), increased lactate (> 2 mmol/L), anemia (hemoglobin ≤ 7.0 g/dL), state of lower adrenal activation (cortisol ≤ 25 μg/dL), cardiogenic shock, absent history of CKD or CLD, in-hospital mortality, and simultaneous measurements of ScvO_2_ and lactate. We completed regression analyses of lactate and ScvO_2_ for patients who were and who were not receiving the lactic acidosis-associated medications. In patients with an SaO_2_ measurement, we also completed a regression analysis of lactate and ScvO_2_ for those who had passed the critical oxygen delivery threshold (oxygen extraction ratio ≥ 50%). We performed additional regression analyses for lactate with factors that could affect lactate levels: cortisol, AST, ALT, total bilirubin, and creatinine. To observe intra-patient correlations between lactate and ScvO_2_, we used a simple linear regression of change in lactate with change in ScvO_2_ for patients with multiple measurements over the course of a single ICU stay. Additionally, we plotted lactate levels vs sextiles of ScvO_2_ and completed a restricted cubic spline analysis of lactate and ScvO_2_ to further describe their relationship. We also performed independent samples *t* tests examining mean lactate levels in patients with CKD and CLD. We also performed multiple linear regression modeling with lactate as the outcome variable and AST, creatinine, ScvO_2_, cortisol, total bilirubin, and the medications of epinephrine, norepinephrine, and linezolid as predictor variables. To determine the contribution to the model of each variable, we calculated the change in *r*^2^ when individual variables were added.

## Results

**Fig. 1:**
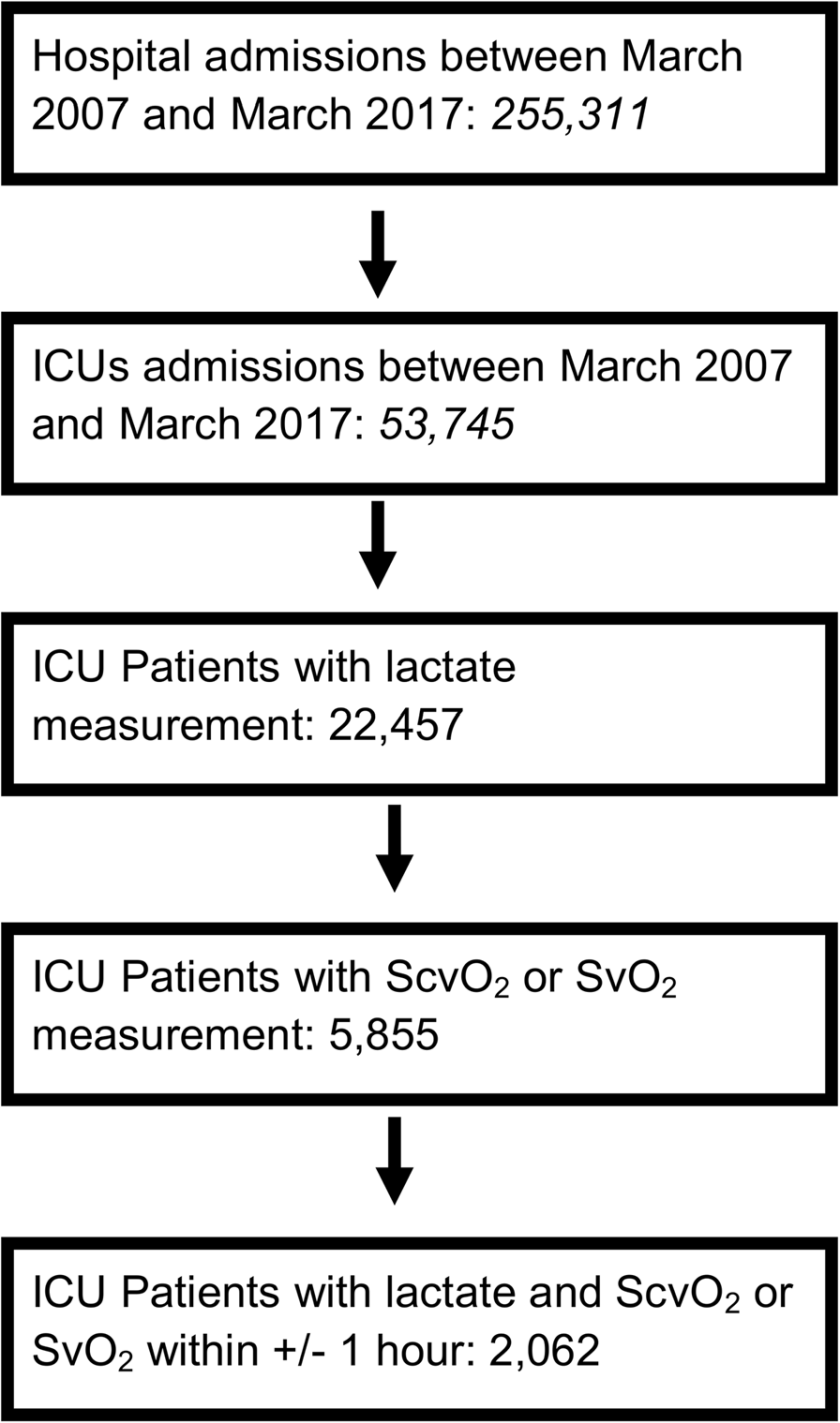
Patient selection flowchart. ICUs = intensive care units. ScvO_2_ = central venous oxygen saturation. SvO_2_ = mixed venous oxygen saturation

During the period of study, a total of 255,311 patients were admitted to the hospital, of which 53,745 patients were admitted to ICUs and 2062 patients were eligible for study, totaling 2348 paired lactate and ScvO_2_ measures (Fig. 1). Demographics and clinical features of the included patients are shown in Table 1. Five hundred seventy-one patients were given an ICD-9 or ICD-10 shock diagnosis, most of them having septic shock (73%). The median serum lactate for the entire cohort was 1.9 mmol/L (IQR 1.3–3.3), and the median ScvO_2_ was 73.5% (IQR 64.5–81.8). There were 1103 increased lactates (> 2 mmol/L), and 607 decreased ScvO_2_ measures (< 65%). The likelihood of ScvO2 < 65% was increased in patients with serum lactate > 2 mmol/L (OR = 1.69, 95% CI = 1.40–2.05, *p* = 0.0000) (Additional file 1).

**Table 1:** Patient demographics and clinical values

Patient characteristics	Value
Mean age, years (SD)	58 (19)
Males (%)	1113 (54)
Mortality (%)	1134 (55)
Mean hospital length of stay (SD)	20 (22)
Mean ICU length of stay (SD)	8.8 (13)
Need for dialysis (%)	309 (15)
Mechanically ventilated (%)	1258 (61)
Sepsis (%)	80 (3.9)
Severe sepsis (%)	1051 (51)
Septic shock (%)	454 (22)
Cardiogenic shock (%)	68 (3.3)
Traumatic shock (%)	49 (2.4)
Shock NOS (%)	43 (2.1)
Diabetes (%)	763 (37)
Chronic kidney disease (%)	701 (34)
Chronic respiratory failure (%)	309 (15)
Chronic hypertension (%)	1340 (65)
Chronic liver disease (%)	639 (31)
Serum lactate < 2.0 mmol/L (%)	1186 (51)
Serum lactate 2.0–4.0 mmol/L	732 (31)
Serum lactate > 4.0 mmol/L (%)	430 (18)
ScvO_2_ < 65 (%)	607 (26)
ScvO_2_ 65–75 (%)	673 (29)
ScvO_2_ > 75 (%)	1068 (45)
Patients receiving epinephrine (%)	206 (10)
Patients receiving norepinephrine (%)	1237 (60)
Patients receiving dopamine (%)	1835 (89)
Patients receiving metformin (%)	134 (6.5)
Patients receiving linezolid (%)	474 (23)
Cortisol mean (SD) in μg/dL (*n* = 603)	37.3 (33.8)
Creatinine mean (SD) in mg/dL (*n* = 1377)	1.87 (1.72)
AST mean (SD) in U/L (*n* = 1166)	374 (1455)
Hemoglobin mean (SD) in g/dL (*n* = 1139)	9.68 (2.22)
Thiamine mean (SD) in μg/dL (*n* = 8)	154 (68.2)
Total bilirubin mean (SD) in mg/dL (*n* = 1170)	2.39 (4.35)

*ICU* ^*i*^ntensive care unit, *ScvO*_*2*_ central venous oxygen saturation, *AST* aspartate aminotransferase

**Fig. 2:**
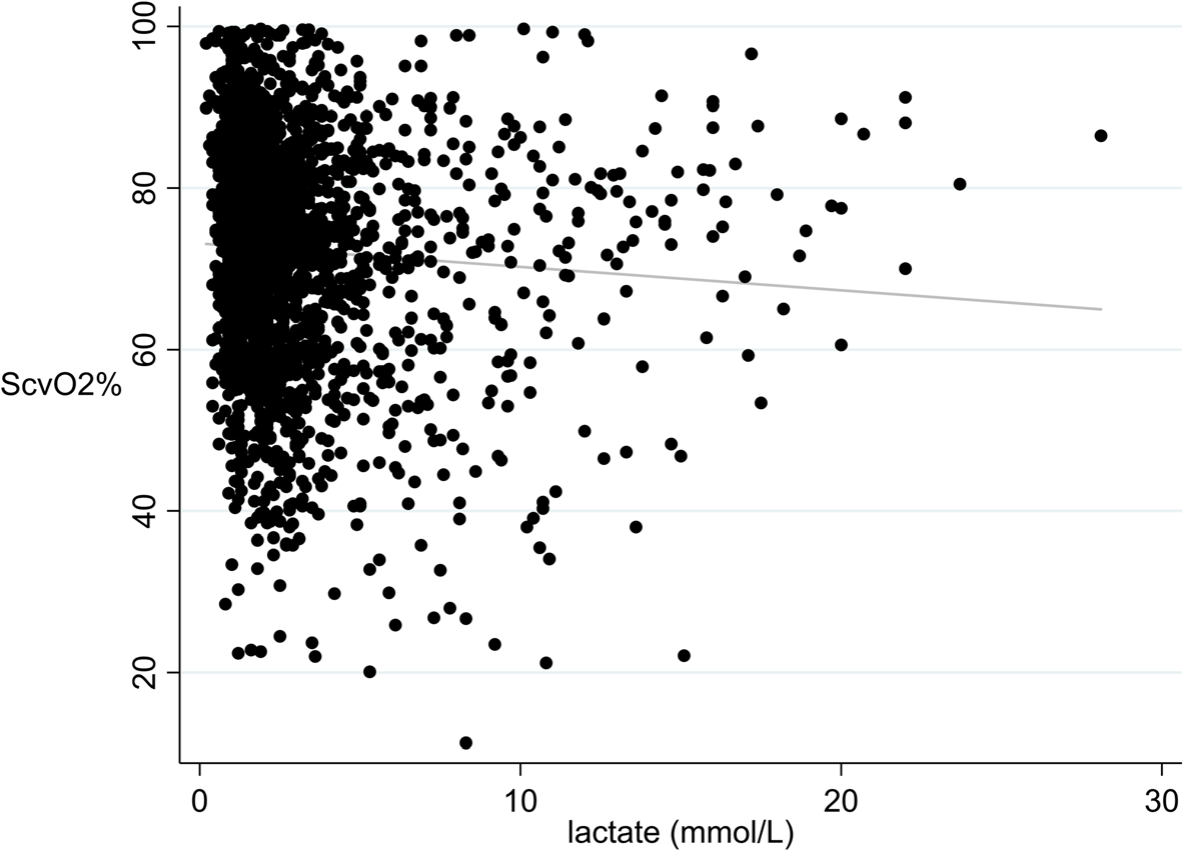
Lactate and ScvO_2_ simple linear regression. ScvO_2_ = central venous oxygen saturation. A simple linear regression between lactate and ScvO_2_ was performed with all pairs of lactate and ScvO_2_ (*n* = 2348) in the study. *r*^2^ = 0.0041 and *p* = 0.0019. The equation for the line of best fit was ScvO_2_ = 73.2–0.29 (lactate)

The simple linear regression data are shown in Additional file 2. We also performed log transformations and exponential analyses, where appropriate, to determine goodness of fit of those models; outcomes displayed similarly weak correlations. Linear regression of lactate and ScvO_2_ showed a weak negative correlation between the two (*n* = 2348, *r*^2^ = 0.0041, *p* = 0.0019) (Fig. 2). We also evaluated lactate and ScvO_2_ simple linear regressions under different conditions. Lactate and ScvO_2_ were not correlated or weakly correlated in patients with presumed tissue hypoxia, as evidenced by lactate > 2 mmol/L (*r*^2^ = 0.0003, *p* = 0.59), high oxygen extraction, indicated by ScvO_2_ ≤ 65% (*r*^2^ = 0.0431, *p* < 0.001), and patients with anemia, indicated by hemoglobin ≤ 7.0 g/dL (*r*^2^ = 0.0062, *p* = 0.41). To mitigate the effect of endogenous epinephrine as a stimulus for lactate production, we evaluated patients with cortisol ≤ 25 μg/dL, as a marker of low adrenal activation, but lactate and ScvO_2_ remained poorly correlated (*r*^2^ = 0.0140, *p* = 0.053). There was no correlation in patients who were not receiving the lactic acidosis-associated medications (*r*^2^ = 0.0317, *p* = 0.25). However, lactate very strongly predicted ScvO_2_ in patients with an oxygen extraction ratio greater than 50% (Additional file 3, *r*^2^ = 0.93, *p* = 0.0019), although only six patients of the 211 with both SaO_2_ and ScvO_2_ measurements had an oxygen extraction ratio of that magnitude. No type of shock predominated in these patients, and liver and kidney functions were intact in all of them. In the restricted cubic spline analysis of lactate and ScvO_2_ (Additional file 4), as well as the lactate vs ScvO_2_ sextile plot (Additional file 5), lactate showed a U-shaped relationship with ScvO_2_, with the highest lactate levels at the lowest and highest percentage values of ScvO_2_.

**Fig. 3:**
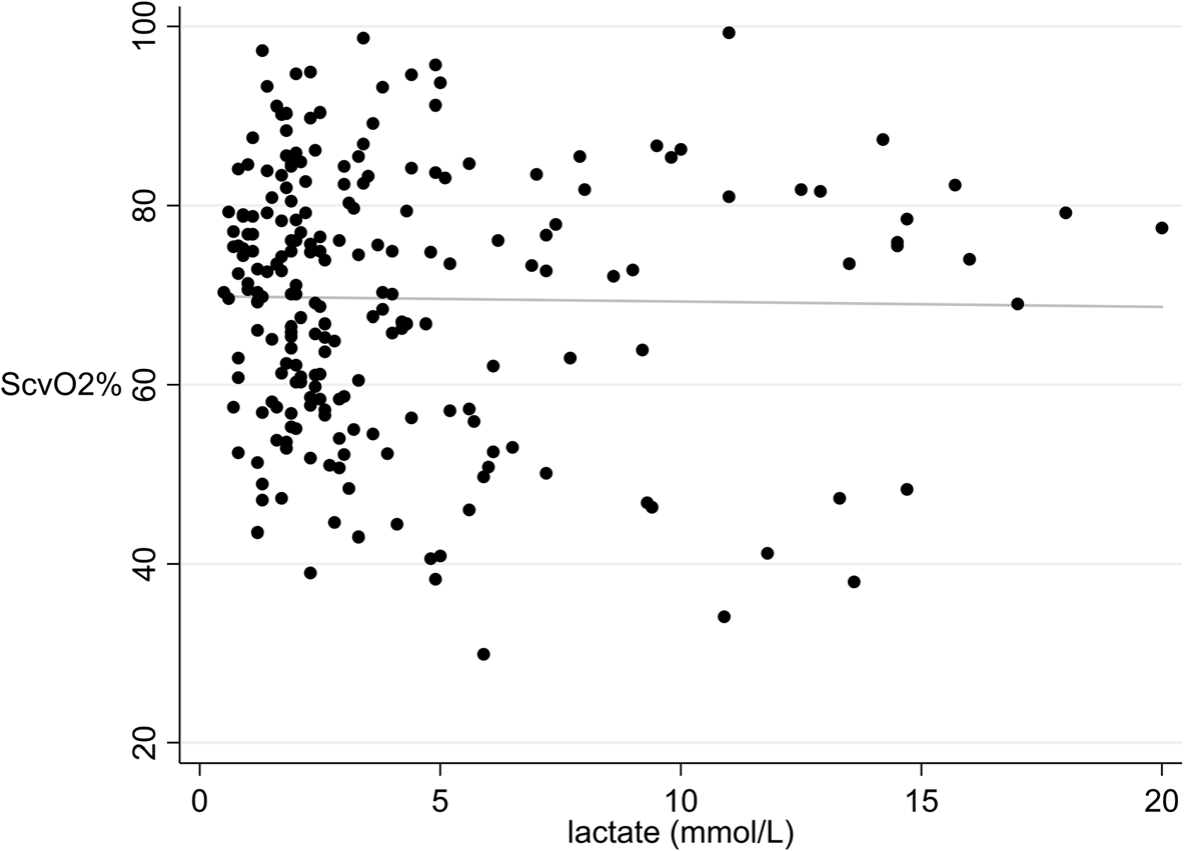
Lactate and ScvO_2_ (only septic shock patients) simple linear regression. ScvO_2_ = central venous oxygen saturation. A simple linear regression was performed with the 508 paired measurements of lactate and ScvO_2_ in patients diagnosed with septic shock. *r*^2^ = 0.0037 and *p* = 0.17. The equation for the line of best fit was ScvO_2_ = 72.7–0.26 (lactate)

Because increased lactate is a significant feature of shock, we assessed lactate and ScvO_2_ regressions in patients with various types of shock. No correlation was found in patients with cardiogenic shock (*r*^2^ = 0.0064, *p* = 0.49) or septic shock (*r*^2^ = 0.0037, *p* = 0.17) (Fig. 3). To examine whether the lack of correlation represented population variability in the relationship of lactate and ScvO_2_, we evaluated intra-patient changes in the two variables among 286 patients who had multiple measurements over the course of an ICU stay; again, the relationship was weak (*r*^2^ = 0.0302, *p* < 0.001), even when we only included patients with some degree of lactate clearance (Additional file 6, *r*^2^ = 0.0137, *p* = 0.19). As described in Additional file 2, lactate and ScvO_2_ displayed weak correlations under conditions of no liver or kidney dysfunction, simultaneous measurement, and only ScvO_2_ values after March 2014. In our analyses with other factors that could affect lactate, it was poorly correlated with cortisol (Additional file 7), AST, ALT (Additional file 8), total bilirubin, creatinine, and the lactic acidosis-associated medications. Patients with CLD had a significantly higher mean lactate than patients without CLD, and there remained only very weak correlation of lactate and ScvO_2_; there was no significant difference in mean lactate when we compared patients with and without CKD (Additional file 9).

Because lactate was a poor predictor of ScvO_2_ in the majority of analyses, we used multiple linear regression modeling to evaluate parameters that may have affected serum lactate levels (Table 2). We evaluated combinations of predictor variables that were statistically correlated with lactate levels in univariate regressions: ScvO_2_, cortisol, creatinine, AST, ALT, total bilirubin, and the medications of epinephrine, norepinephrine, and linezolid. Table 2 shows the effect of any one of these variables in the model by showing the effect on *r*^2^ of removing the indicated variable from the model. The most significant model had a coefficient of determination (*r*^2^) of 0.3667 (*p* < 0.001) and included all predictor variables. ScvO_2_, on average, added only 0.001 to the *r*^2^ of any multiple linear regression model.

**Table 2:** Selected multiple linear regressions predicting lactate

Variable removed from full model	*n*	*r* ^2^	*p* value
Full model^a^	319	0.3667	< 0.001
Epinephrine	327	0.3325	< 0.001
Norepinephrine	327	0.3462	< 0.001
Linezolid	327	0.3486	< 0.001
Total bilirubin	351	0.3350	< 0.001
ALT	327	0.3547	< 0.001
Creatinine	351	0.3503	< 0.001
Cortisol	885	0.2310	< 0.001
ScvO_2_	327	0.3544	< 0.001
AST	347	0.2286	< 0.001

^a^Full model: AST, ALT, ScvO_2_, cortisol, creatinine, total bilirubin, medications (epinephrine, norepinephrine, linezolid)

## Discussion

Reductions in ScvO_2_ occur when oxygen delivery to tissues is reduced and oxygen extraction is increased [8]. Lactate, the product of anaerobic metabolism, is considered the biomarker of choice for determining the presence of tissue hypoxia [1–3, 5, 9]. Recent critical care literature, including clinical practice guidelines, intertwines these two biomarkers as indicative of “tissue hypoperfusion,” an entity which is not clearly defined, but which represents some combination of reduced tissue oxygen delivery and tissue hypoxia [30, 31]. Since lactate and ScvO_2_ are both well-studied independent predictors of mortality in sepsis [17–23], their relationship should be clarified, because of their frequent use as interchangeable markers of improving tissue oxygenation [30, 31]. Furthermore, both lactate and ScvO_2_ are used as resuscitation markers in cardiogenic and hemorrhagic shock [24–28]. However, ScvO_2_ can be either increased or decreased in sepsis, while lactate has been considered an indicator of tissue oxygenation, due to its association with total oxygen debt [15, 16, 22].

To our knowledge, this is the first direct analysis of the relationship between serum lactate levels and central venous oxygen saturation in critically ill patients, and the most comprehensive, although Weil evaluated the relationship of mixed venous oxygen saturation, oxygen delivery, and lactic acidosis [29]. Our data both complement and confirm those of Gattinoni et al., which demonstrate a complex relationship between lactate and ScvO_2_ [32]. In our single-center, retrospective, cohort study, lactate strongly predicted ScvO_2_ when patients were at or below the critical oxygen delivery threshold, but only 3% of shock patients met this criterion. In the overall population of critically ill patients, serum lactate predicted ScvO_2_ poorly, even after controlling for patients with high adrenal activation, normoxia, or kidney and liver dysfunction, all of which could modulate lactate levels via alternative mechanisms. Furthermore, when the analysis was limited to patients with increased lactate, or to patients with low ScvO_2_, there was no meaningful relationship between the two variables.

To evaluate the relationship of lactate and ScvO_2_ under conditions of potentially reduced oxygen delivery, we performed simple linear regression correlations of lactate and ScvO_2_ under conditions of presumed tissue hypoxia (lactate > 2 mmol/L), high oxygen extraction (ScvO_2_ ≤ 65%), anemia (hemoglobin < 7.0 g/dL), septic shock, and cardiogenic shock. Weak correlations between the two variables were observed with all of these conditions, suggesting the presence of other factors or combinations of factors that contributed to increased lactate. While the odds of having a decreased ScvO_2_ (< 65%) were 1.69 times greater if lactate was increased (> 2 mmol/L), only 31.1% of patients with an increased lactate had a decreased ScvO_2_. These numbers offer little support for using lactate and ScvO_2_ as interchangeable markers of “tissue perfusion.” Recognizing that lactate and ScvO_2_ values can change rapidly, we evaluated simultaneous lactate and ScvO_2_ measurements, but the correlation remained weak. A previous study indirectly observed similar relationships, as 79% of septic patients without lactate clearance had simultaneous ScvO_2_ > 70% [17]. Furthermore, low ScvO_2_ values can be observed in the presence of either high or low lactate levels [18]. Our serial measurements of simultaneous lactate and ScvO_2_ also support that the relationship of lactate and ScvO_2_ is inconsistent even within individual patients, further implicating the influence of factors other than tissue hypoxia on lactate levels.

One factor that may have led to the weak correlation between lactate and ScvO_2_ is the release of endogenous epinephrine during stress, leading to lactate production through beta-adrenergic receptor stimulation [33, 34]. We used serum cortisol as a surrogate marker for epinephrine, since epinephrine levels are not routinely measured in critically ill patients. Stress responses during critical illness stimulate the adrenal cortex to release cortisol and catecholamines, which correlate with the degree of stress experienced [35]. Although cortisol did not correlate strongly with serum lactate levels, it was a more consistent predictor of lactate than were tissue oxygenation markers.

Hepatic and renal dysfunction, common complications of shock and sepsis, can also reduce lactate elimination, potentially further contributing to the lack of correlation between lactate and the assessed markers. Lactate is metabolized by the liver and excreted by the kidney, so dysfunction of either organ could reduce lactate elimination [36, 37]. Our study showed that patients with liver dysfunction, but not kidney dysfunction, have significantly increased lactate levels. However, in the linear regressions, we observed only weak correlations between lactate and markers of hepatic or renal dysfunction. Our findings corroborate a previous study correlating lactate clearance with total bilirubin or renal creatinine clearance, which also did not demonstrate a significant relationship [38].

To mitigate the effect of alternate sources of lactate in critical illness on the relationship between lactate and ScvO_2_, we performed regressions under multiple constraints. However, lactate and ScvO_2_ were weakly correlated under conditions of low adrenal activation, absence of acute or chronic hepatic and renal dysfunction, or absence of lactic acidosis-associated medications. Additionally, the multiple linear regression model does not fully explain the association of these variables, evidenced by low *r*^2^ values, suggesting that additional factors outside the scope of our investigations affected serum lactate levels. AST contributed the largest increase to the *r*^2^, suggesting that acute liver injury was the factor with the greatest predictive value for serum lactate.

It has been proposed that the inverse relationship of lactate and ScvO_2_ exists only below the critical oxygen delivery threshold (oxygen extraction ratio ≥ 50%) [8, 39]. However, only six of a possible 211 patients in our study exhibited an O_2_ extraction ratio of this magnitude. While a simple linear regression of lactate and ScvO_2_ in those patients displayed a strong correlation, it seems clear that relatively few shock patients may actually be at or below the critical threshold, suggesting that in the vast majority of patients, lactate and ScvO_2_ will neither accurately reflect one another, nor will lactate adequately reflect tissue hypoxia. Lactate and ScvO_2_ may be inversely correlated under certain conditions, but these conditions are unlikely to be present in most shock patients.

The predominantly weak correlation between lactate and ScvO_2_ demonstrates the complexity of lactate as a biomarker above the critical oxygen delivery threshold. Lactate consumption by the brain and heart is increased during stress, and lactate is released by the lungs during sepsis, which support that oxygen delivery is not the only factor, and perhaps not the major one, influencing serum lactate levels [40]. Increased lactate in critical illness is likely to be caused by a combination of factors, and the extent and timing of them may vary significantly from patient to patient. Additionally, even though lactate tends to increase at advanced stages of illness, ScvO_2_ can either increase or decrease, further complicating the relationship of lactate and markers of tissue hypoxia [23]. These factors most likely contributed to the weak correlations between lactate and the variables included in this study. However, our study does support that lactate and ScvO_2_ are correlated in the small subset of patients who have passed the critical oxygen delivery threshold. Therefore, in the vast majority of critically ill patients, lactate is primarily affected by factors other than tissue hypoxia and should not be used as a predictor of ScvO_2_ or tissue oxygenation.

Our study has some weaknesses, including that the findings are limited to a single center, the analysis is retrospective, and some aspects of the data are confirmatory of previous observations. Reduced thiamine levels can also be associated with increased lactate, but we were unable to account for thiamine levels, as these are rarely measured in our ICUs. While only 30% of the patient population had a shock diagnosis based on the ICD codes, this is likely due to well-reported discrepancies between administrative data and correct diagnoses [41]. In our institution, combined lactate and ScvO_2_ measurements are made only in patients with shock. We also combined ScvO_2_ and SvO_2_ measurements in the regressions, which could have altered the distribution of values. However, SvO_2_ should be strongly correlated with ScvO_2_, even if not equal to it, and lactate was also shown to have little correlation with the known ScvO_2_ and SvO_2_ values after March 2014. Lastly, we were not able to correlate lactate levels with endogenous epinephrine levels since epinephrine is not routinely measured. We used cortisol as a surrogate for epinephrine, but the relationship between cortisol and epinephrine levels is not fully understood and may not be consistent.

This study does have multiple strengths, including its large number of paired measurements and its evaluation of the intra-patient lactate/ScvO_2_ relationships. We also evaluated multiple shock states, organ dysfunctions, and pre-existing conditions, as well as conditions of presumed tissue hypoxia, low oxygen delivery, and increased oxygen extraction. Previous studies examining oxygen delivery have either assumed that increased lactate is equivalent to passing the critical O_2_ delivery point or have been achieved in patients being terminally removed from life support; neither of these circumstances is of clinical utility [8, 42]. Our data suggest that very few shock patients (approximately 3%) have reached critical levels of O_2_ delivery. Finally, we were able to test the within-patient direction and magnitude of change of lactate and ScvO_2_ values.

## Conclusions

Based on the results of this single-center retrospective cohort study, lactate has very little predictive ability for ScvO_2_ in the vast majority of critically ill patients, even after accounting for factors that could interfere with such a relationship. In the clinical setting, lactate should not be used interchangeably with ScvO_2_ as a marker of tissue hypoxia.

## Additional files

Additional file 1: Cross-tabulation table of lactate > 2 mmol/L and ScvO_2_ < 65%. Table displaying numbers of patients with increased or decreased lactate and concurrent increased or decreased ScvO_2._ (DOCX 12 kb)

Additional file 2: Selected Simple Linear Regression Data. Simple Linear Regressions involving serum lactate. (DOCX 14 kb)

Additional file 3: Lactate and ScvO_2_ Simple Linear Regression in patients with Oxygen Extraction Ratio ≥ 50%. There were six patients who had both SaO_2_ and ScvO_2_ to allow the calculation of the Oxygen Extraction Ratio ((SaO_2_ – ScvO_2_)/SaO_2_). A lactate and ScvO_2_ simple linear regression in these patients produced an r^2^ of 0.9303, *p* = 0.0019. The equation for the line of best fit was ScvO_2_ = -6.23(lactate) +59.2. (TIF 347 kb)

Additional file 4: Restricted Cubic Spline Analysis of lactate and ScvO_2._ Three knots are inserted to describe the relationship of lactate and ScvO_2_. Only lactate levels < 10 mmol/L were used in this analysis. (TIF 1065 kb)

Additional file 5: Lactate vs ScvO_2_ sextiles. Lactate plotted vs ScvO_2_ sextiles. Data are presented as mean ± standard error. (TIF 287 kb)

Additional file 6: Change in lactate vs Change in ScvO_2_ or SvO_2_ in patients with lactate clearance. Of the patients with multiple measurements of lactate and ScvO_2_ or SvO_2_ over the course of an ICU stay, there were 130 patients who had some level of lactate clearance (lactate difference > 0 mmol/L). The r^2^ for the change in lactate vs change in ScvO_2_ or SvO_2_ regression for this population was 0.0137, *p* = 0.19. The equation for the line of best fit was ScvO_2_ or SvO_2_ difference = -0.82(lactate difference) – 0.66. (TIF 415 kb)

Additional file 7: Lactate and Cortisol Simple Linear Regression. There were 603 measurements of lactate and cortisol in the appropriate time frame. r^2^ = 0.0627 and the *p* < 0.001. The equation for the line of best fit is Cortisol = 28.83 + 2.39(lactate). (TIF 504 kb)

Additional file 8: Lactate and ALT Simple Linear Regression. Only ALT values less than 100 U/L were included in the graph. There were 935 measurements of lactate and ALT (<100 U/L) in the appropriate time frame. r^2^ = 0.011 and the *p* = 0.0013. The equation for the line of best fit is ALT = 0.93(lactate) + 27.0. (TIF 1716 kb)

Additional file 9: Independent Samples T Tests for lactate in patients with CKD or CLD. Tables displaying independent samples t test summaries for lactate levels in patients with CKD or CLD. (DOCX 12 kb)

## Data Availability

The datasets used and/or analyzed during the current study are available from the corresponding author on reasonable request.

## Abbreviations

ALT: Alanine aminotransferase
AST: Aspartate aminotransferase
CKD: Chronic kidney disease
CLD: Chronic liver disease
CRF: Chronic respiratory failure
EMR: Electronic medical record
ICD-10: International Classification of Diseases, Tenth Revision
ICD-9: International Classification of Diseases, Ninth Revision
ICUs: Intensive care units
LOS: Length of hospital stay
LOS-ICU: Length of ICU stay
NOS: Not otherwise specified
ScvO_2_: Central venous oxygen saturation
SvO_2_: Mixed venous oxygen saturation
TUKH: The University of Kansas Hospital in Kansas City, Kansas
