# Supplementary material for: Serum lactate poorly predicts central venous oxygen saturation in critically ill patients: a retrospective cohort study
Source: J Intensive Care. 2019 Sep 5;7:47. doi: 10.1186/s40560-019-0401-5 (PMC6728973; doi:10.1186/s40560-019-0401-5)
Supplement: Supplementary file 1 — Cross-tabulation table of lactate > 2 mmol/L and ScvO2 < 65%. Table displaying numbers of patients with increased or decreased lactate and concurrent increased or decreased ScvO2. (DOCX 12 kb) [file 40560_2019_401_MOESM1_ESM.docx]

**Additional File 1**

|  | **Lactate ≥ 2 mmol/L** | **Lactate < 2 mmol/L** | **Total** |
| --- | --- | --- | --- |
| **ScvO_2_ < 65%** | 344 | 263 | 607 |
| **ScvO_2_ ≥ 65%** | 759 | 982 | 1,741 |
| **Total** | 1,103 | 1,245 | 2,348 |

Additional File 1: Cross-tabulation table of lactate > 2 mmol/L and ScvO_2_ < 65%

*^a^*Central Venous Oxygen Saturation
