# Supplementary material for: Serum lactate poorly predicts central venous oxygen saturation in critically ill patients: a retrospective cohort study
Source: J Intensive Care. 2019 Sep 5;7:47. doi: 10.1186/s40560-019-0401-5 (PMC6728973; doi:10.1186/s40560-019-0401-5)
Supplement: Supplementary file 2 — Selected Simple Linear Regression Data. Simple Linear Regressions involving serum lactate. (DOCX 14 kb) [file 40560_2019_401_MOESM2_ESM.docx]

**Additional File 2**

Additional File 2: Selected Simple Linear Regression Data

|  | Simple Linear Regression | n | r^2^ | p-value |
| --- | --- | --- | --- | --- |
| 1 | Lactate and ScvO_2_*^a^* (ScvO_2_ ≤ 65%) | 613 | 0.0431 | <0.001 |
| 2 | Lactate and ScvO_2_ (hemoglobin ≤ 7.0 g/dL) | 111 | 0.0062 | 0.41 |
| 3 | Lactate and ScvO_2_ (cortisol ≤ 25 μg/dL) | 268 | 0.0140 | 0.053 |
| 4 | Lactate and ScvO_2_ (cardiogenic shock) | 77 | 0.0064 | 0.49 |
| 5 | Lactate and ScvO_2_ (AST*^b^* ≤ 40 U/L and creatinine ≤ 1.2 mg/dL) | 284 | 0.0517 | <0.001 |
| 6 | Lactate and ScvO_2_ (ALT*^c^* ≤ 50 U/L and creatinine ≤ 1.2 mg/dL) | 340 | 0.0284 | <0.002 |
| 7 | Lactate and ScvO_2_ (not receiving lactic-acidosis associated medications) | 44 | 0.0317 | 0.25 |
| 8 | Lactate and ScvO_2_ (doesn’t have CKD*^d^* or CLD*^e^*) | 1,031 | 0.0051 | 0.022 |
| 9 | Lactate and ScvO_2_ (in-hospital mortality) | 1,296 | 0.0009 | 0.28 |
| 10 | Change in lactate and change in ScvO_2_*^f^* | 286 | 0.0302 | <0.001 |
| 11 | Lactate and ScvO_2_ (simultaneous measurements) | 1,548 | 0.0119 | <0.001 |
| 12 | Lactate and ScvO_2_ (cardiogenic shock and lactate ≥ 2 mmol/L) | 46 | 0.0104 | 0.50 |
| 13 | Lactate and ScvO_2_ (lactate > 2 mmol/L) | 1,103 | 0.0003 | 0.59 |
| 14 | Lactate and AST | 1,166 | 0.1402 | <0.001 |
| 15 | Lactate and ALT | 1,186 | 0.0965 | <0.001 |
| 16 | Lactate and total bilirubin | 1,170 | 0.0278 | <0.001 |
| 17 | Lactate and creatinine | 1,377 | 0.0262 | <0.001 |
| 18 | Lactate and epinephrine (medication) | 2,348*^f^* | 0.0659 | <0.001 |
| 19 | Lactate and metformin (medication) | 2,348*^f^* | 0.0000 | 0.96 |
| 20 | Lactate and norepinephrine (medication) | 2,348*^f^* | 0.0069 | <0.001 |
| 21 | Lactate and linezolid (medication) | 2,348*^f^* | 0.0024 | 0.020 |
| 22 | Lactate and dopamine (medication) | 2,348*^f^* | 0.0006 | 0.24 |
| 23 | Lactate and known ScvO_2_ values*^g^* | 737 | 0.0086 | 0.012 |

*^a^*Central Venous Oxygen Saturation; *^b^*Aspartate Aminotransferase; *^c^*Alanine Aminotransferase; *^d^*chronic kidney disease; *^e^*chronic liver disease; *^f^*The change in lactate and change in ScvO_2_ for patients with multiple measurements of lactate and ScvO_2_ over the course of an ICU stay; *^g^*The regression includes patients receiving and not receiving the medication; *^g^*These are the known ScvO_2_ values after March 2014
