# Supplementary figures and images for: Serum lactate poorly predicts central venous oxygen saturation in critically ill patients: a retrospective cohort study
Source: J Intensive Care. 2019 Sep 5;7:47. doi: 10.1186/s40560-019-0401-5 (PMC6728973; doi:10.1186/s40560-019-0401-5)

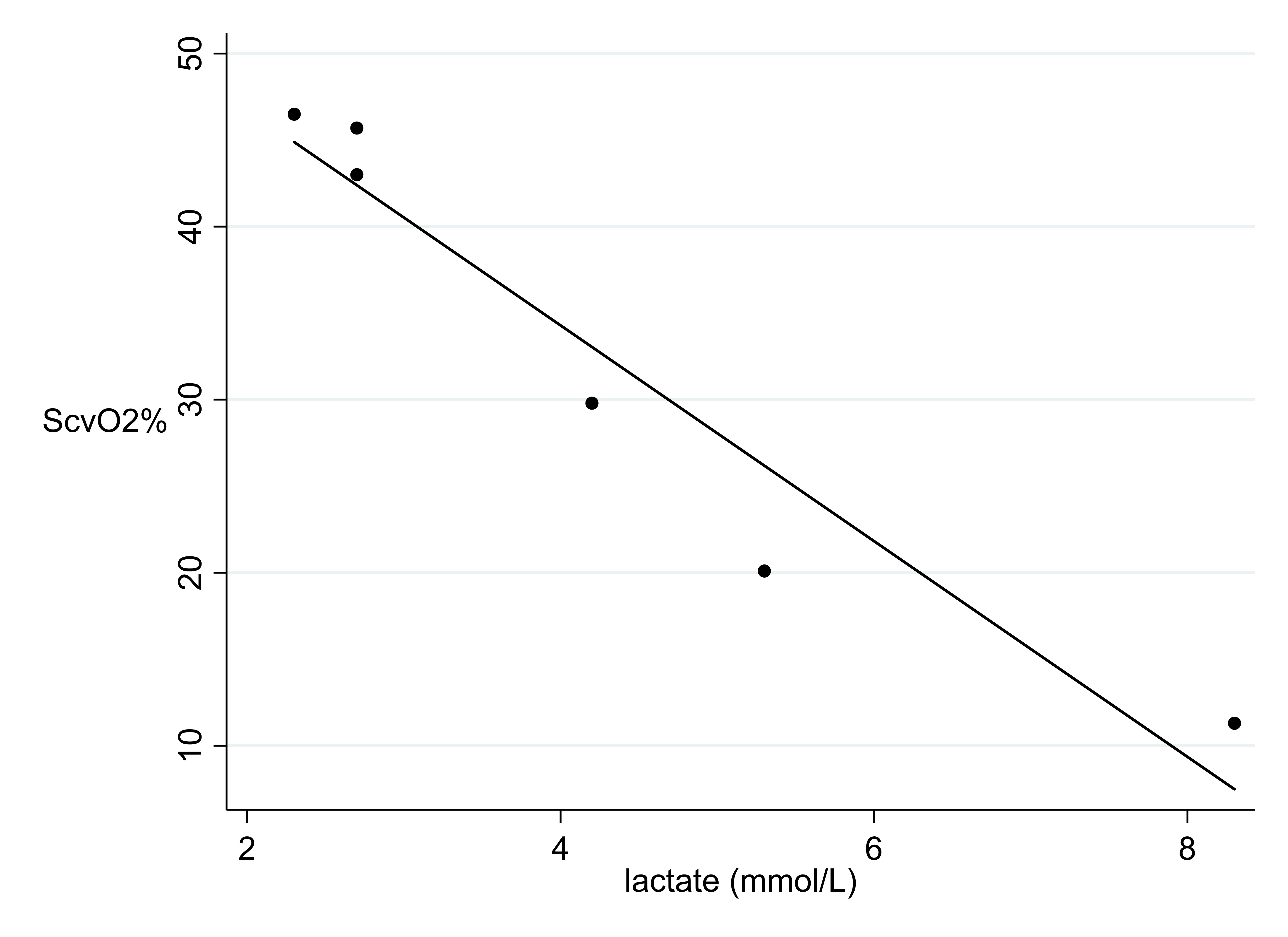

Supplement: Supplementary file 3 — Lactate and ScvO2 Simple Linear Regression in patients with Oxygen Extraction Ratio ≥ 50%. There were six patients who had both SaO2 and ScvO2 to allow the calculation of the Oxygen Extraction Ratio ((SaO2 – ScvO2)/SaO2). A lactate and ScvO2 simple linear regression in these patients produced an r2 of 0.9303, p = 0.0019. The equation for the line of best fit was ScvO2 = -6.23(lactate) +59.2. (TIF 347 kb) [file 40560_2019_401_MOESM3_ESM.tif]

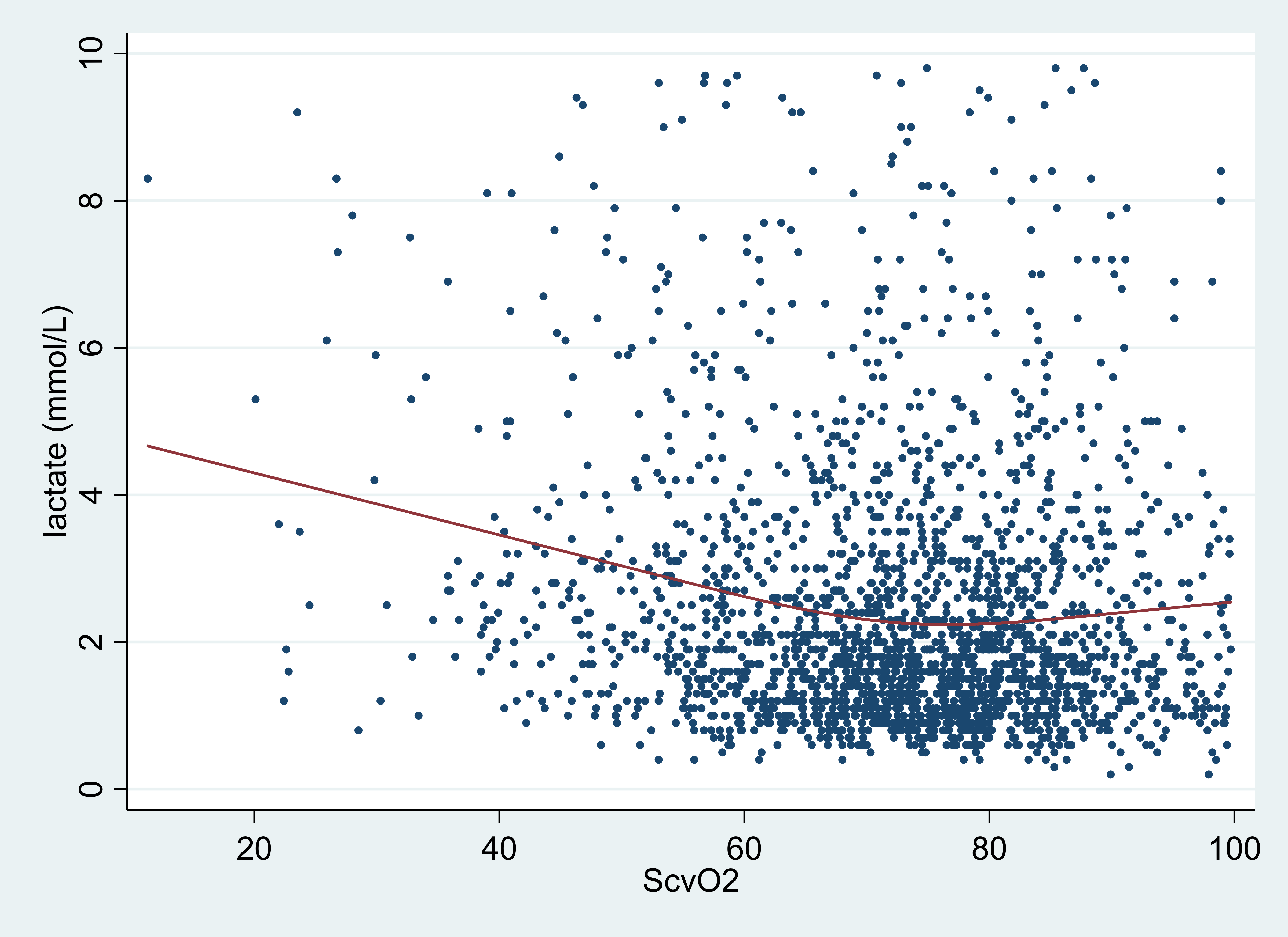

Supplement: Supplementary file 4 — Restricted Cubic Spline Analysis of lactate and ScvO2. Three knots are inserted to describe the relationship of lactate and ScvO2. Only lactate levels < 10 mmol/L were used in this analysis. (TIF 1065 kb) [file 40560_2019_401_MOESM4_ESM.tif]

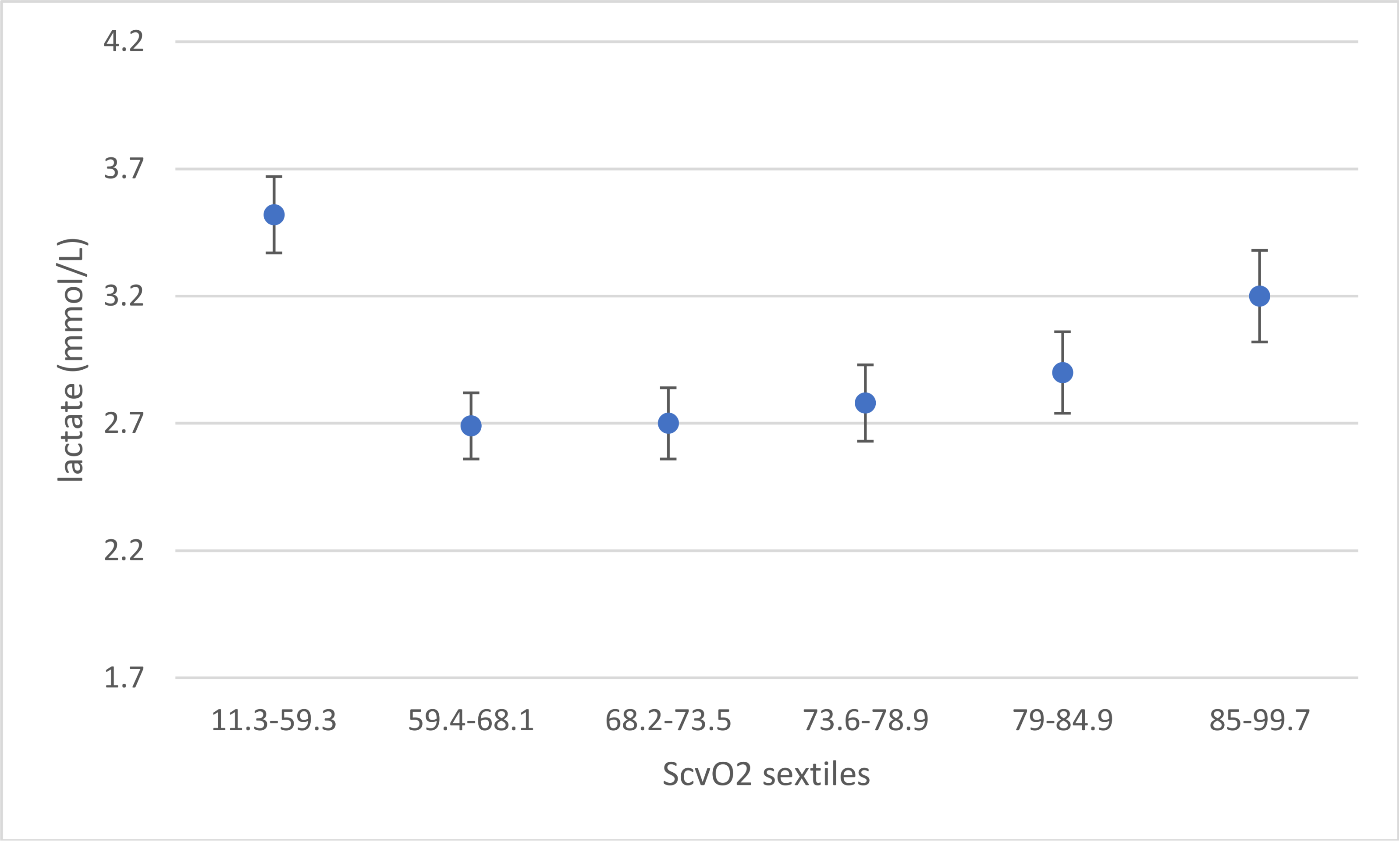

Supplement: Supplementary file 5 — Lactate vs ScvO2 sextiles. Lactate plotted vs ScvO2 sextiles. Data are presented as mean ± standard error. (TIF 287 kb) [file 40560_2019_401_MOESM5_ESM.tif]

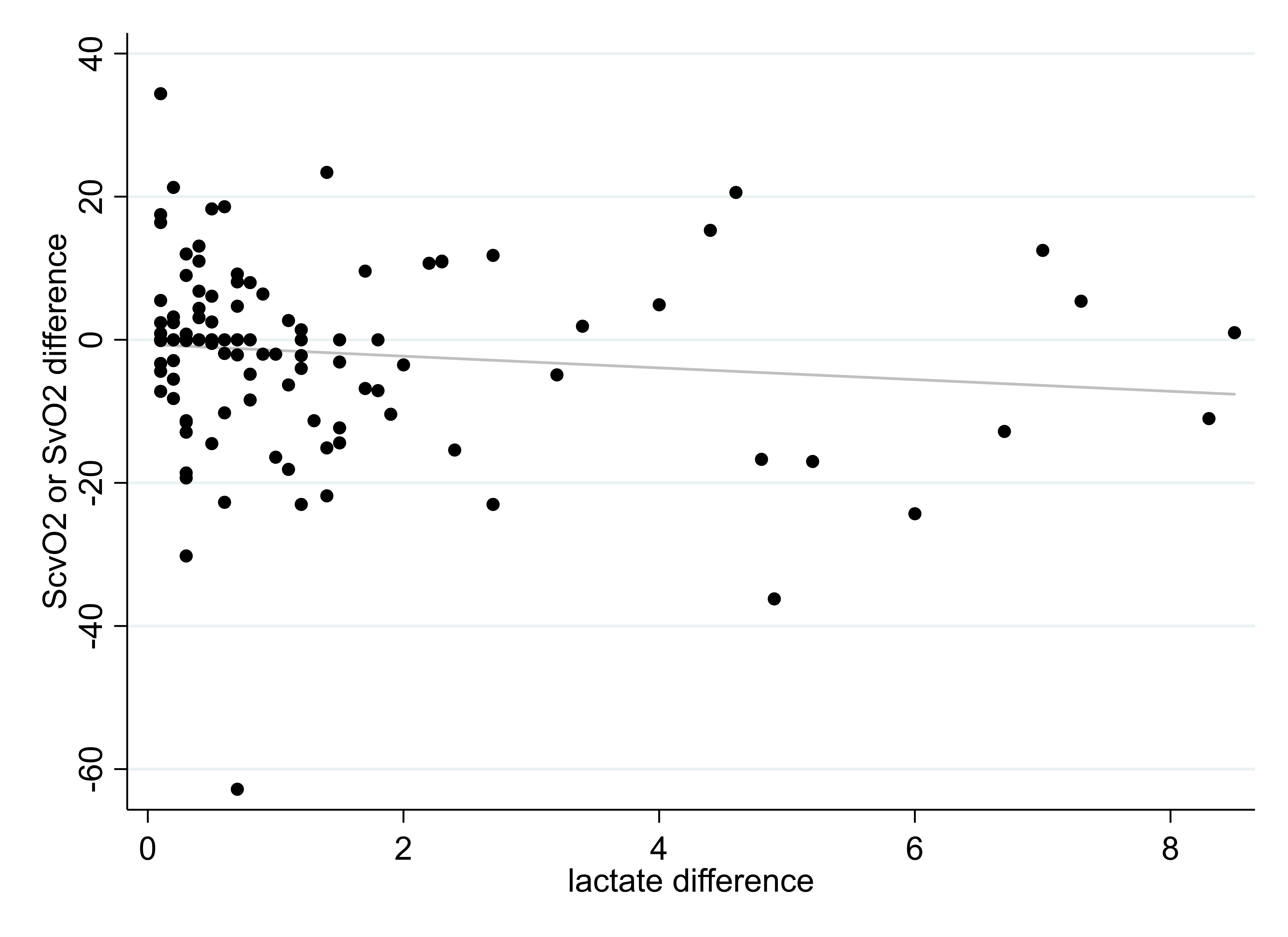

Supplement: Supplementary file 6 — Change in lactate vs Change in ScvO2 or SvO2 in patients with lactate clearance. Of the patients with multiple measurements of lactate and ScvO2 or SvO2 over the course of an ICU stay, there were 130 patients who had some level of lactate clearance (lactate difference > 0 mmol/L). The r2 for the change in lactate vs change in ScvO2 or SvO2 regression for this population was 0.0137, p = 0.19. The equation for the line of best fit was ScvO2 or SvO2 difference = -0.82(lactate difference) – 0.66. (TIF 415 kb) [file 40560_2019_401_MOESM6_ESM.tif]

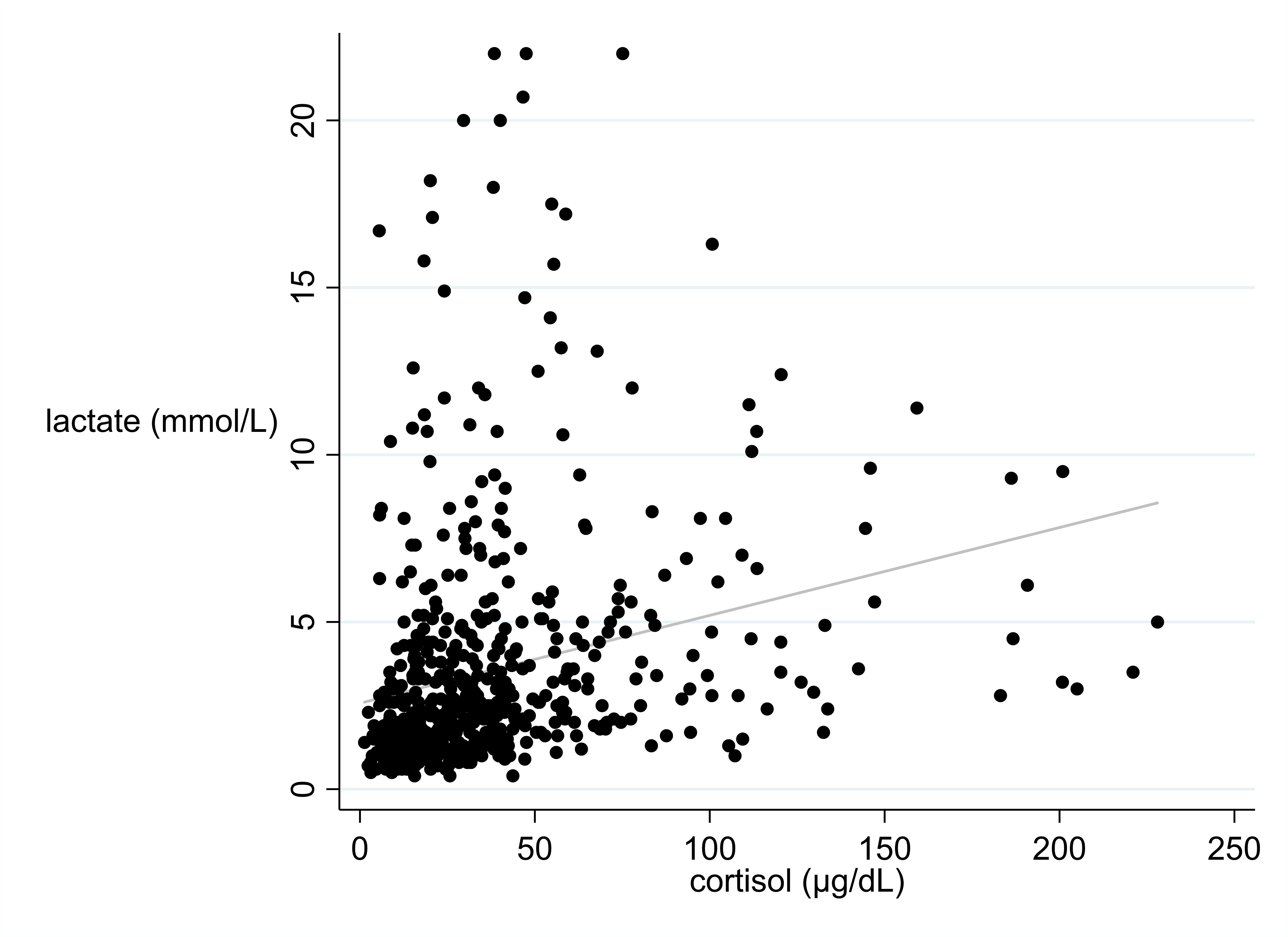

Supplement: Supplementary file 7 — Lactate and Cortisol Simple Linear Regression. There were 603 measurements of lactate and cortisol in the appropriate time frame. r2 = 0.0627 and the p < 0.001. The equation for the line of best fit is Cortisol = 28.83 + 2.39(lactate). (TIF 504 kb) [file 40560_2019_401_MOESM7_ESM.tif]
