# Supplementary material for: Serum lactate poorly predicts central venous oxygen saturation in critically ill patients: a retrospective cohort study
Source: J Intensive Care. 2019 Sep 5;7:47. doi: 10.1186/s40560-019-0401-5 (PMC6728973; doi:10.1186/s40560-019-0401-5)
Supplement: Supplementary file 9 — Independent Samples T Tests for lactate in patients with CKD or CLD. Tables displaying independent samples t test summaries for lactate levels in patients with CKD or CLD. (DOCX 12 kb) [file 40560_2019_401_MOESM9_ESM.docx]

**Additional File 9**

Additional File 9: Independent Samples T Tests for lactate in patients with CKD or CLD

**Independent Samples T Test for lactate in patients with Chronic Liver Disease (CLD):**

|  | Mean Lactate (mmol/L) | Standard Error in lactate (mmol/L) | 95% Confidence Interval |
| --- | --- | --- | --- |
| Patients without CLD | 2.54 | 0.065 | [2.40, 2.67] |
| Patients with CLD | 3.84 | 0.14 | [3.57, 4.11] |

T = -8.54 p < 0.001

**Independent Samples T Test for lactate in patients with Chronic Kidney Disease (CKD):**

|  | Mean Lactate (mmol/L) | Standard Error in lactate (mmol/L) | 95% Confidence Interval |
| --- | --- | --- | --- |
| Patients without CKD | 3.02 | 0.084 | [2.85, 3.18] |
| Patients with CKD | 2.85 | 0.097 | [2.66, 3.04] |

T = 1.31 p = 0.19
